# Proteomic Analysis and Identification of Possible Allergenic Proteins in Mature Pollen of *Populus tomentosa*

**DOI:** 10.3390/ijms19010250

**Published:** 2018-01-16

**Authors:** Liuqiang Wang, Xiaoling Zhang, Jin Zhang, Wei Fan, Mengzhu Lu, Jianjun Hu

**Affiliations:** 1 State Key Laboratory of Tree Genetics and Breeding, Key Laboratory of Tree Breeding and Cultivation of the State Forestry Administration, Research Institute of Forestry, Chinese Academy of Forestry, Beijing 100091, China; wanglq@caf.ac.cn (L.W.); xlzhang@126.com (X.Z.); zhang007jin@163.com (J.Z.); atom201001@163.com (W.F.); lumz@caf.ac.cn (M.L.); 2 Co-Innovation Center for Sustainable Forestry in Southern China, Nanjing Forestry University, Nanjing 210037, China

**Keywords:** allergen, MALDI-TOF/TOFMS/MS, pollen, *Populus tomentosa*, two-dimensional gel electrophoresis

## Abstract

Pollen grains from *Populus tomentosa*, a widely cultivated tree in northern area of China, are considered to be an important aeroallergen causing severe allergic diseases. To gain insight into their allergenic components, mature *Populus tomentosa* pollen proteins were analyzed by two-dimensional gel electrophoresis (2-DE) and matrix-assisted laser desorption/ionization-time of flight mass spectrometry (MALDI-TOF/TOF MS). A total of 412 spots from mature pollen were resolved on pH 4–7 immobilized pH gradient (IPG) strips and 159 distinct proteins were identified from 242 spots analyzed. The identified proteins were categorized based on their functional role in the pollen, which included proteins involved in energy regulation, protein fate, protein synthesis and processing, metabolism, defense/stress responses, development and other functional categories. Moreover, among the identified proteins, 27 proteins were identified as putative allergens using the Structural Database of Allergenic Proteins (SDAP) tool and Allergen Online. The expression patterns of these putative allergen genes indicate that several of these genes are highly expressed in pollen. The identified putative allergens have the potential to improve specific diagnosis and can be used to develop vaccines for immunotherapy against poplar pollen allergy.

## 1. Introduction

Pollen grains are the dispersal agents of sperm cells and play a vital role in sexual reproduction of higher plants. After release from an anther, pollen grains are carried by wind, insects and other agents to the stigma of a carpel, with the primary function of delivering sperm cells to the female gametophyte via the formation of a pollen tube, and subsequent seed and fruit production [1,2]. During the development of pollen, microsporogenesis and microgametogenesis, a large number of genes are coordinated and expressed in different tissues of an anther with designated roles in cell signaling, cytoskeleton formation, cell wall metabolism and vesicle transport [3,4]. Pollen grains have proteins from 2.5% to 61% by dry mass and some of those proteins act as allergens upon inhalation [5]. To date, a large amount of information on pollen allergens in diverse plant species can be found in allergen databases such as the Allergome [6] or Structural Database of Allergenic Proteins (SDAP) [7]. However, pollen allergens are restricted to few protein families and show distinct patterns of species distribution; the major pollen allergen families are composed of profilins, expansins and calcium-binding proteins [8]. In recent years, the incidence and prevalence of pollen allergy has increased worldwide, with deleterious implications for health, such as asthma and allergic rhinitis in sensitized subjects [9,10].

With the completion of many plant species genome sequences and availability of comprehensive public sequence databases, proteomics investigations of pollen have developed rapidly. For example, Dai et al. [11] identified several novel proteins in rice pollen that may be involved in signal transduction, protein synthesis, assembly and degradation, and wall remodeling and metabolism. Proteomic analysis of tomato pollen showed that many of the identified proteins have designated roles in defense mechanisms, energy conversion, pollen germination, and pollen tube growth, and some possibly in sperm cell formation [2]. More recently, Zou et al. [12] reported comparative proteomic analysis of Arabidopsis mature pollen and germinated pollen, and indicated some differentially expressed proteins that are mainly involved in different cellular and metabolic processes, including cell signaling, cellular structure, transport, defense/stress responses, transcription, metabolism and energy production.

In recent years, proteomic technologies have been increasingly employed in the field of allergy, allowing the identification of new allergens by using two-dimensional gel electrophoresis (2-DE) coupled with mass spectrometry (MS) [13,14]. For instance, Akagawa et al. [15] identified flour wheat allergens (serine protease inhibitors (serpin), α-amylase inhibitor, ç-gliadin and low-molecular-weight (LMW) glutenin) and their sequentially homologous proteins as major IgE (Immunoglobulin E)-binding proteins using the allergenomic approach. Mani et al. [14] first identified seven IgE-reactive proteins from *L*. *lucidum* pollen by 2-DE immunoblotting and MS. *Cocos nucifera* (Coconut) pollen proteins were analyzed and within them were identified 12 allergenic proteins by 2-DE, immunoblotted with coconut pollen sensitive patient sera followed by mass spectrometry of IgE-reactive proteins [16]. In addition, Saha et al. [17] first identified ten novel allergens in *Phoenix sylvestris* pollen using de novo sequencing and homology-based proteomics technologies.

The genus *Populus* contains approximately 30 species of woody plants and is widely distributed in the Northern hemisphere and is exhibiting some of the fastest growth rates observed for trees growing in temperate climates [18]. In spring, *Populus* releases large amounts of pollens and these pollens are considered to be an important aeroallergen causing severe allergic diseases in an increasing percentage of hypersensitive individuals. However, research on poplar pollen allergy is limited to its clinical manifestations and basic immunochemical studies. *Populus tomentosa* is a poplar species and is widely cultivated in northern area of China for shelterbelts and urban afforestation. To understand the allergenic composition and obtain information about allergens from *P. tomentosa* pollen, we isolated the total protein from mature pollen using the trichloroacetic acid and acetone (TCA-A) method and identified candidate allergenic proteins by 2-DE and MS. Furthermore, the expression patterns of the identified possible allergenic proteins were analyzed in the different tissues. As far as we know, this is the first report of comprehensive proteomic analysis and possible allergenic proteins in mature pollen of *P. tomentosa*.

## 2. Results and Discussion

### 2.1. In Vitro Pollen Germination

**Figure 1:**
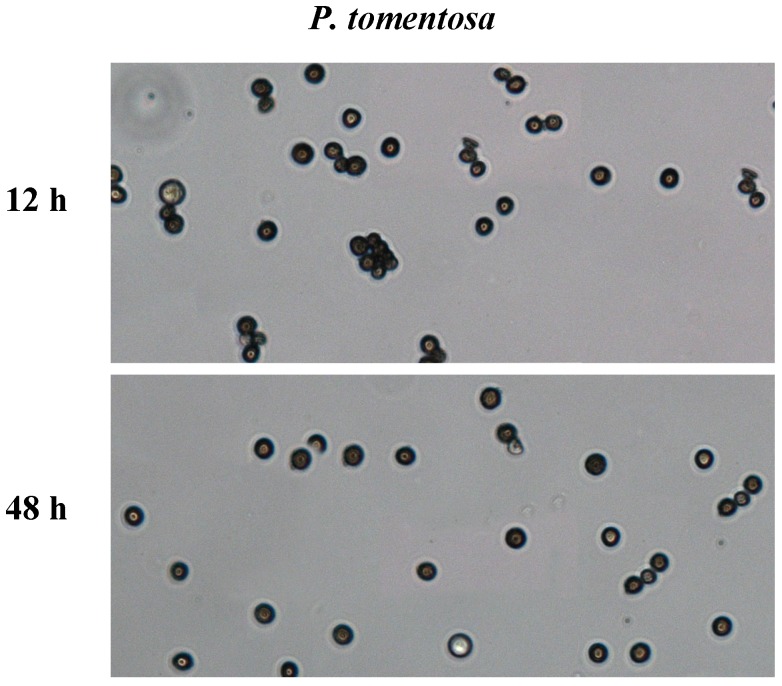
Pollen germination rate of *P. tomentosa*. The pollen grain suspension with the liquid germination medium was spread on the dishes. After incubation at 21 °C for 12 and 48 h, the pollen germination rate was determined under a light microscope. The experiments were repeated three times and three replicates (dishes) were carried out.

Mature pollen grains were harvested from *P. tomentosa* and sowed on dishes containing liquid germination medium using the “thin liquid layer” germination method. At the same time, the pollen of triploid poplar ‘ZhongHuai1’ (‘ZH1’) and diploid poplar ‘ZhongHuai2’ (‘ZH2’) were conducted in parallel. The pollen of ‘ZH2’ began to germinate after 2 h cultivation and the germination rate was increased with the extending of incubation time. After incubation at 21 °C for 48 h, over 88% pollen of diploid ‘ZH2’ was germinated, while all of the pollen of triploid ‘ZH1’ was not germinated [19]. Consistent with the pollen of triploid ‘ZH1’, the mature pollen grains of *P. tomentosa* were not germinated at all, indicating that the fast-growing triploid of *P. tomentosa* is sterile (Figure 1).

### 2.2. Proteomic Maps of P. tomentosa Mature Pollen

**Figure 2:**
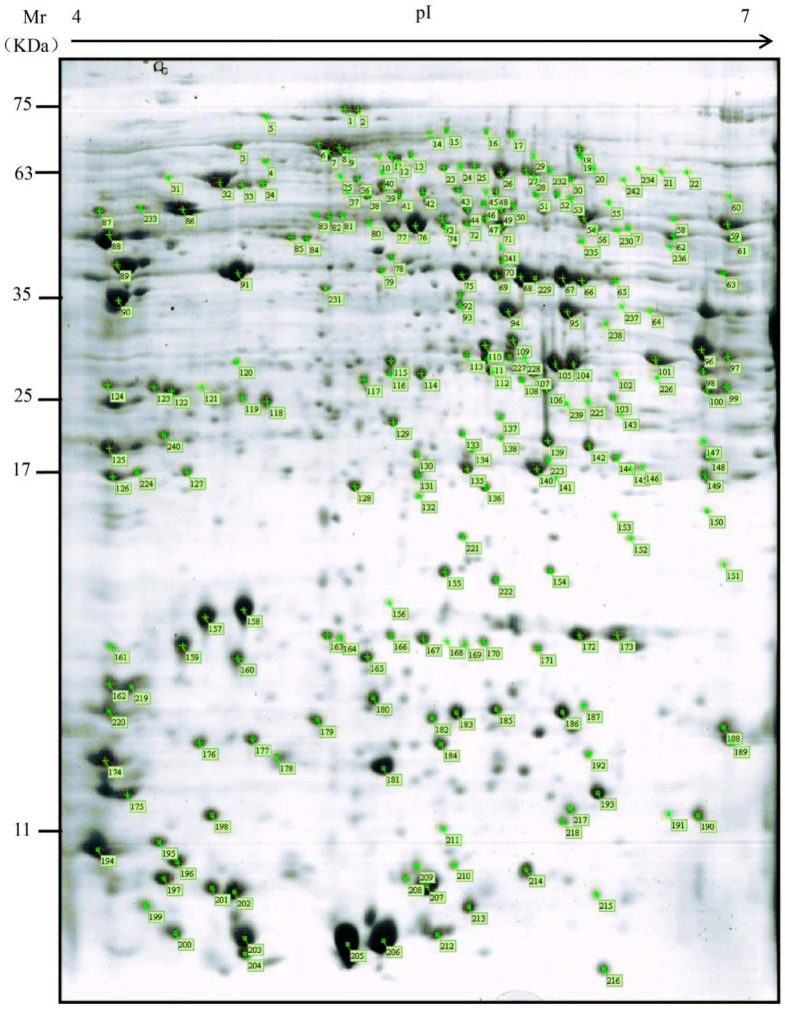
Colloidal coomassie stained 2-DE gel of *P. tomentosa* mature pollen. Spot numbers indicated on the gel were subjected to MALDI-TOF/TOF MS/MS analysis. Standard molecular weight markers are shown on the left. All spots with protein identifications are listed in Table S1.

Total proteins extracted from mature *P. tomentosa* pollen were subjected to 2-DE stained with CCB using pH 4–7 IPG strips, then the 2-DE gels were aligned and matched. A total of 412 reproducible protein spots were detected in the gels and were resolved at the molecular weight (*M*_W_) range from 5 to 120 kDa and isoelectric point (p*I*) values range from 4 to 7 (Figure 2). All detected protein spots were processed by automated in gel tryptic digestion and MALDI-TOF/TOF MS/MS analysis. 242 protein spots, representing 159 different proteins, were subjected to BLAST against the *Populus trichocarpa* proteome (v3.0), which was downloaded from the Phytozome 10.3.1 website [20] However, it is noted that there were 83 proteins associated with different spots. The calculated *M*_W_ of the identified proteins ranged from approximately 8.94 to 199.37 kDa and p*I* values range from approximately 4.11 to 12.21, the PACid number and corresponding *P. trichocarpa* gene locus were also listed in Table S1. Most of them are close to the experimental data as judged from the location of the spots on the 2-DE gels. However, it should be noted that the identified proteins did not always have a one-to-one correlation with the spots on the gels and the deviations in molecular mass and p*I*, which may have resulted from a number of factors. For example, polypeptide variants that present in different spots on the gel, but encoded by the same gene, post-translational modification of the proteins in vivo (e.g., phosphorylation, glycosylation, acetylation, methylation or other groups) that do not significantly affect the *M*_W_ of a protein but induce a p*I* shift on the protein spot on the gel [21,22,23], protein translation from alternatively spliced mRNAs [24], partial synthesis of proteins during pollen maturation [25] or chemical modification of the proteins during sample preparation.

### 2.3. Functional Classification of Identified Proteins

**Figure 3:**
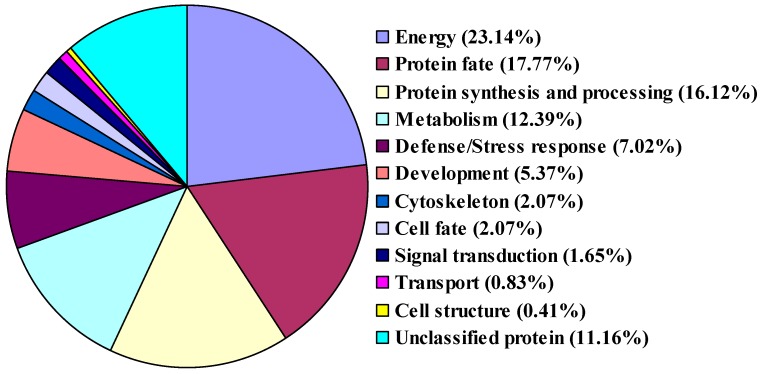
Functional annotation of identified *P. tomentosa* pollen proteins. The percentage values indicate the proportion of total number of proteins within that category.

To assign functional information to the identified proteins, we classified them into functional categories according to the gene sequences and a homologic comparison with other known proteins [2,12,26]. The 242 identified proteins were classified into 12 different functional groups (Figure 3, Table S1). Approximately 70% of them were classified into four categories, including energy (23.14%), protein fate (17.77%), protein synthesis and processing (16.12%) and metabolism (12.39%), suggesting a special requirement of these categories of proteins for energy and general metabolism, such as protein synthesis, oxidative phosphorylation, carbohydrate metabolism and sugar metabolism. These proteins were also reported in rice and Arabidopsis pollen [1,11,23]. Although the presence of a high percentage of proteins related to energy metabolism correlates well with the large number of mitochondria observed in mature *P. tomentosa* pollen, it is well known that the pollen germination and tube growth are high-energy-requiring processes that require most of the proteins for these processes, the *P. tomentosa* mature pollen was not germinated. The other functional categories were defense/stress responses (7.02%), development (5.37%), cytoskeleton (2.07%), cell fate (2.07%), signal transduction (1.65%), transport (0.83%), cell structure (0.41%) and unclassified proteins (11.16%). There were 27 proteins out of the 242 that could not be functionally classified as they were not observed to contain any known conserved domains.

**Figure 4:**
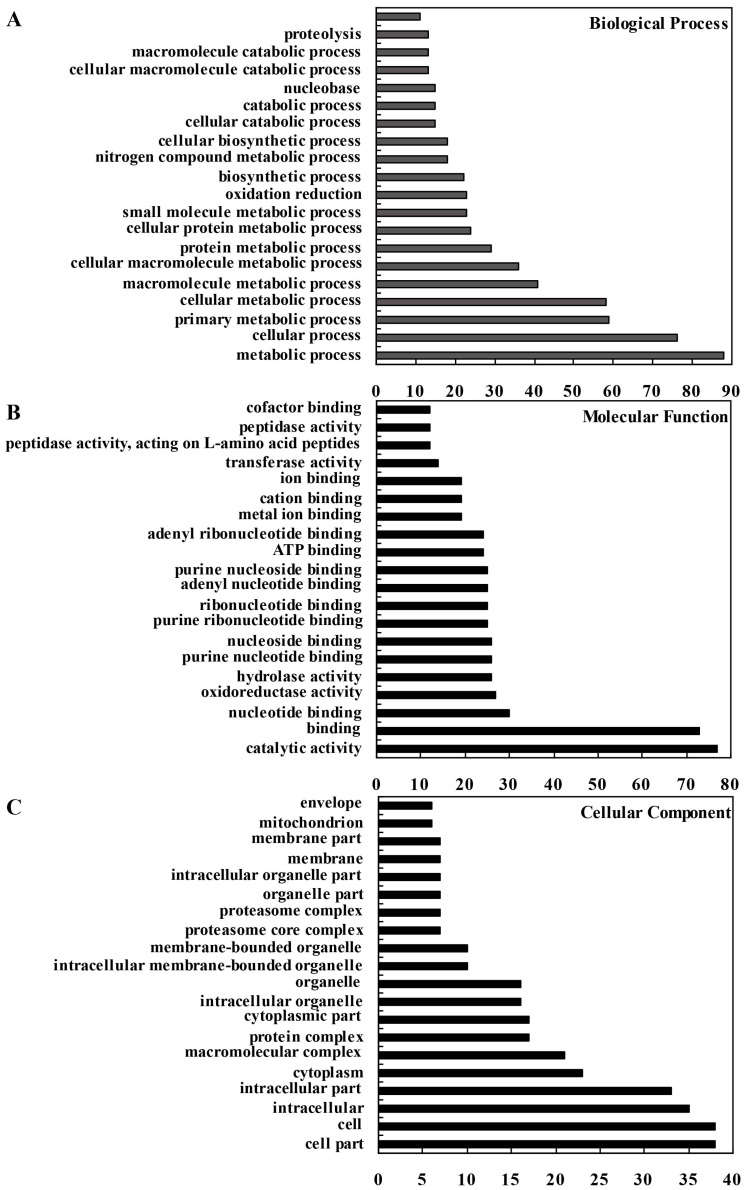
Gene ontology analysis of identified proteins. Results are summarized for three main GO categories, including biological process (**A**); molecular function (**B**) and cellular component (**C**). The number corresponding to each column indicates the number of proteins in this GO term.

In addition, gene ontology (GO) assignments were performed to functionally classify these proteins, which provide a dynamic, controlled vocabulary, and hierarchical relationships for the representation of information on the biological process, molecular function and cellular component (Figure 4). In terms of biological process, metabolic process (GO: 0008152, 88 proteins) was the most represented GO term, followed by cellular process (GO: 0009987, 76 proteins) and primary metabolic process (GO: 0044238, 59 proteins). In molecular function, proteins with catalytic activity (GO: 0003824, 77 proteins) and binding (GO: 0005488, 73 proteins) were highly represented. Regarding cellular component, the most represented categories were cell part (GO: 0044464, 38 proteins), cell (GO: 0005623, 38 proteins), intracellular (GO: 0005622, 35 proteins) and intracellular part (GO: 0044424, 33 proteins).

### 2.4. Prediction of Allergens in P. tomentosa Mature Pollen

Pollens are one of the leading causes of respiratory allergic sensitizations [27]. In spring, poplars release a lot of pollen that might cause the allergenic response. To date, many sequences and structures of pollen allergenic proteins have been characterized and restricted to few protein families [8]. They share common characteristics that contribute to their ability to bind IgE and trigger an allergic reaction [28]. To identify the potential allergen proteins in *P. tomentosa* mature pollen, the 242 identified proteins were predicted with SDAP tool and Allergen Online. SDAP is a web server that can provide rapid, cross-referenced access to the sequences, structures and IgE epitopes of allergenic proteins [7], and Allergen Online is a better and more frequently updated website. In this study, 27 proteins identified in poplar pollen were predicted as putative allergens (Table 1). For example, eleven heat shock protein 70 (Hsp70) (Spots 4, 5, 7, 8, 9, 10, 11, 12, 13, 21 and 31) and four small Hsps (spots 172, 173, 217 and 218) were identified as corresponding to allergenic molecules. Hsp70 have been demonstrated to bind to human IgE from allergenic patients to cystic echinococcosis [29], corn and barley [30], and antigenic cross-activity of Hsp70 with a 70 kDa component was proved by amino acid sequence alignment in *Penicillium citrinum* [31]. Class I small heat shock protein (Hsp) detected on a 2D gel have reported that it is one of allergens in soybean [32]. Spots 25, 49, 50, 53, 54, 59, 62, 71, 72, 222, 241 correspond to enolase. It is a ubiquitous glycolytic enzyme that was observed as highly conserved allergens from various fungi and latex, such as *Cladosporium herbarum* (Cla h 6), *Alternaria alternate* (Alt a 6), *Curvularia lunata* (rCur l 2) [33,34]. Spots 156, 159 and 160 were identified as pollen Ole e 1 allergen, which was a well characterized allergenic protein with the relevant (24–34%) homologous amino acid sequence among pollen proteins from maize, tomato, ryegrass, birch, rice, Arabidopsis etc., and was surmised to control pregermination and pollen tube emergence [35]. Spots 55 and 210 were identified as thioredoxin, which is known to act as a novel cross-reactive cereal allergen family that might contribute to the symptoms of baker’s asthma and might be related to grass pollen allergy [36]. Weichel et al. [36] identified wheat thioredoxin hB (Tri a 25) by screening a cDNA phage display library against immobilized serum IgE from 8 bakers with occupational asthma. It shared high homology with maize thioredoxin (ZmTRXh1 and ZmTRXh2) and human thioredoxin and included cross-reactive members that might be of relevance for patients occupationally exposed to inhalant allergens. Spot 197 and 202 respectively correspond to profilin 3 and 5. Profilins are ubiquitous proteins in the vegetal kingdom that act as pan-allergens, and are actin-binding proteins present in all eukaryotic cells. The family of profilin is one of the main causes of cross-reactivity between pollen and vegetable food [37], and their clinical allergenicity, albeit variable, is well recognized both in respiratory and food allergy [38]. Plant profilins present a highly conserved structure that provokes multiple positive sIgE responses in sensitized patients [39]. Spot 145 correspond to triosephosphate isomerase, which described as allergen in wheat, latex and lychee [40] and the remaining allergenic proteins predicted caused allergic reactions still need to be further studied. More importantly, the IgE antibody binding properties of these allergenic proteins should be analyzed using immunoblotting with sera from patients with pollen allergy. This could have confirmed the IgE recognition of the putative allergens as well as confirmed the cross-reactivity to pollen allergens from other species [40]. In our previous study, we identified 28 possible allergenic proteins in *P. deltoides* CL. ‘2KEN8’ [26]. Here, we compared the overlap of these candidate possible allergenic proteins between *P. tomentosa* and ‘2KEN8’ (Figure S1) and the results showed that 16 possible allergenic proteins were present in both *P. tomentosa* and ‘2KEN8’ mature pollen, such as Hsp, enolase, pollen Ole e 1, profilin and thioredoxin. In addition to the 16 allergenic proteins, they have 23 different putative allergens. These differences may be due to the methods of protein extraction, spot selection for analysis and the poplar species. 

### 2.5. Expression Profiles of the Predicted Allergen Genes in Different Tissues

**Figure 5:**
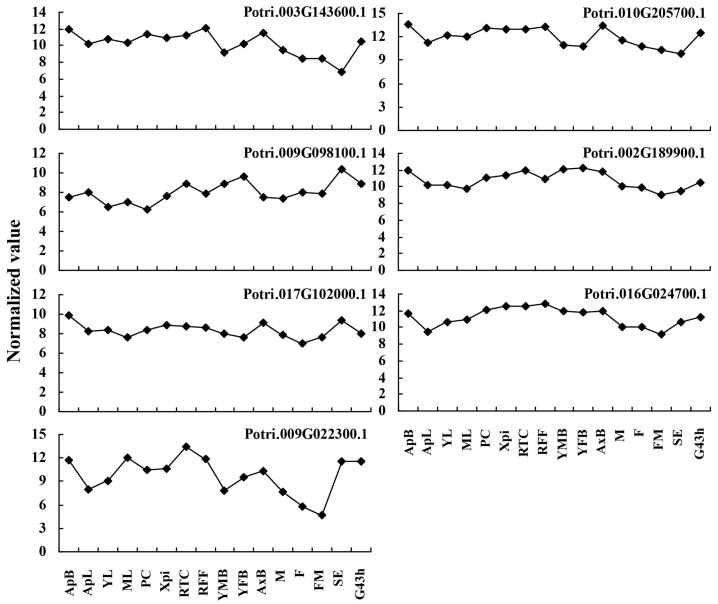
Expression profiles of genes coding predicted allergen proteins across different tissues. The Affymetrix microarray data (accession number GSE21481) were obtained from NCBI Gene Expression Omnibus (GEO) database. ApB, Shoot apex; ApL, Young leaves at apex; YL, Young leaves plastochron #2; ML, Mature leaves plastochron #5; PC, Phloem and cortex; Xpi, Developing xylem and pith; RTC, Roots from tissue culture; RFF, Roots from field trees; YMB, Male floral bud initials; YFB, Female floral bud initials; AxB, Axillary buds; M, Male catkin-3 stages pooled; F, Female catkin post-pollination; FM, Mature catkin before seed release; SE, Seed; G43 h, Seedling 43 h post-imbibition.

To examine whether the predicted pollen-allergen genes presently characterized are expressed in poplar and to study their expression patterns, Zhang et al. [26] previously showed that the global expression patterns of 28 predicted poplar allergen genes (including 16 putative allergen genes presented in both *P. tomentosa* and ‘2KEN8’ mature pollen) across various tissues based on an Affymetrix microarray data (GSE21481). Among the 16 putative allergen genes, two genes (Potri.001G392400.1 and Potri.011G111300.1) corresponding to spots 156, 159 and 160 (Pollen Ole e 1 allergen and extensin family protein) had high transcript levels in male catkin, suggesting their specific expression in pollen. In this study, we analyzed the expression patterns of the 11 putative allergen genes that only presented in *P. tomentosa* across various tissues based on this microarray data. However, five poplar allergen genes (Potri.005G015100.1, Potri.013G009500.1, Potri.012G090900.1 and Potri.013G089200.1) did not agree with the corresponding data. The reasons may be due to the improvement of the poplar genome, some genes with the incorrect functional annotation have been removed or others. The expression patterns of the other 7 poplar allergen genes are shown in Figure 5. Three poplar allergen genes corresponding to spots 9 (Potri.010G205700.1, Hsp 70 family protein), 48 (Potri.002G189900.1, Aldehyde dehydrogenase 2B7) and 194 (Potri.016G024700.1, Calmodulin 6) had high transcript levels in the different tissues. One gene (Potri.003G143600.1) corresponding to spots 4, 8 and 21 (Hsp 70 family protein) was highly expressed in RFF and AxB, the other gene (Potri.009G022300.1) corresponding to spots 215 (Cystatin B) was highly expressed in ApB, ML, RTC, RFF, SE and G43h. These potential allergenic protein genes might play important roles in not only reproduction but also vegetative development. Thus, our data contribute to the identification of new pollen allergenic proteins.

**Figure 6:**
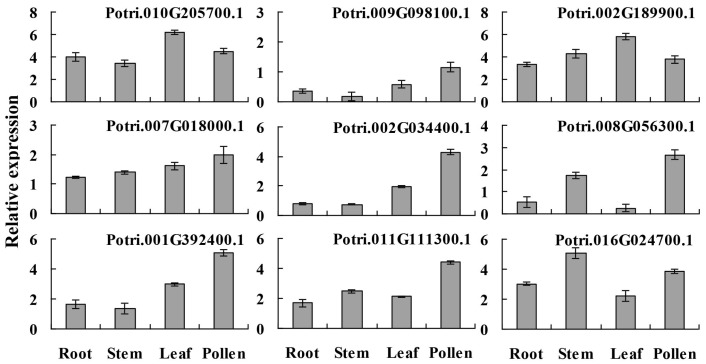
Expression patterns of putative allergen genes in different tissues. Nine predicted allergen protein genes were randomly selected and the expression levels in leaf, stem, root and pollen were analyzed using qRT-PCR. The error bars were calculated from three replicates.

To further confirm the expression profiles of the presently characterized predicted allergen genes and verify the reliability of the microarray data, qRT-PCR analysis was performed on root, stem, leaf and pollen for 9 genes, which had high relative expression levels based on microarray data (Figure 6). Meanwhile, the root, stem and leaf were used as control tissues for study the tissue specific expression. In this study, qRT-PCR results show that the Potri.001G392400.1 and Potri.011G111300.1 corresponding to spots 156, 159 and 160 (Pollen Ole e 1 allergen and extensin family protein) were highly expressed in pollen, and microarray data show that these genes were highly expressed in male catkin [26]. Three poplar putative allergen genes (Potri.010G205700.1, Potri.002G189900.1 and Potri.016G024700.1) had high transcript levels in the different tissues. However, some spots were not consistent with microarray data, the reasons may be that pollen used in qRT-PCR was purer than the male catkin used in the microarray analysis in tissue level, different poplar species and others. In general, the present qRT-PCR results were in good agreement with the microarray data sets analyzed in this study.

## 3. Materials and Methods

### 3.1. Plant Materials and Pollen Isolation

For biological replicates, three uniformly developed flowering branches were collected from one genotype of *P. tomentosa* in a nursery of Chinese Academy of Forestry, and then transferred to buckets filling with water and cultured in a greenhouse at average temperature of 22 °C with a relative humidity of 70–75% under 16 h light/18 h darkness photocycle conditions. Mature pollen grains were collected from freshly anther-dehisced flowers by shaking the tassel on a glass petri dish, dried at 37 °C, and any debris removed with a needle. Pollen samples were used immediately or pooled in a tube, then frozen in liquid nitrogen and stored at −80 °C until further study.

### 3.2. In Vitro Pollen Germination Assay

The viability of each pollen sample was tested using the “thin liquid layer” germination methods with some modifications [41]. Briefly, pollen grains were sowed on the dishes containing the liquid germination medium and incubated at 21 °C in the dark. The liquid germination medium was composed of 15% (*w*/*v*) sucrose, 100 mg/L H_3_BO_3_, 300 mg/L CaCl_2_, 200 mg/L MgSO_4_, 100 mg/L KNO_3_, and the pH was adjusted to 6.0. After incubation for 2, 4, 8, 12, 24, and 48 h, the rates of pollen grains were calculated to determine under a light microscope when the pollen tube grows longer than the diameter of the pollen grain. Each sample was observed in 5 fields of view. At least 30 pollen grains were analyzed in each field. The experiment was repeated three times and three replicates (dishes) were carried out.

### 3.3. Preparation of Total Protein Extraction

Total soluble protein from mature pollen was isolated using the trichloroacetic acid and acetone (TCA-A) method with slight modifications [26]. Briefly, the pollen grains were ground in liquid nitrogen into fine powder and transferred to cold protein extraction buffer containing 10% (*w*/*v*) TCA and 0.07% (*v*/*v*) β-mercaptoethanol in acetone, incubated overnight at −20 °C, and then centrifuged at 14,000 *g* at 4 °C for 30 min. The precipitate was washed three times with the same cold protein extraction buffer without 10% (*w*/*v*) TCA, followed by incubation at −20 °C for 1 h and subsequent centrifugation at 14,000 *g* at 4 °C for 30 min for each wash. The resulting pellets were vacuum-dried, weighed and stored at −80 °C for further use. Each experiment was carried out by three biological replicates.

### 3.4. Two-Dimensional Gel Electrophoresis (2-DE)

The vacuum-dried protein samples were dissolved in a lysis buffer containing 7 M urea, 65 mM dithiothreitol (DTT), 4% (*w*/*v*) CHAPS, 2 M thiourea, and 0.2% carrier ampholytes for 1 h at room temperature with vortexing every 10 min, the homogenate was centrifuged at 15,000 *g* for 20 min. The protein concentration of the supernatant was determined by Bradford assay with bovine serum albumin as the standard [42]. 2-DE was performed following the protocol described by Sheoran et al. [1] and Zhang et al. [26]. Briefly, Protein samples (600 µg) were diluted in a rehydration buffer for 12 h. Isoelectric focusing (IEF) was performed using the Ettan III system (GE Healthcare, Chicago, IL, USA) and 18 cm Immobiline Dry Strips (pH 4–7, GE Healthcare). After IEF, the strips were treated in an equilibration buffer, placed on top of the vertical sodium dodecyl sulfate-polyacrylamide gel electrophoresis (SDS-PAGE) gels and sealed with agarose and bromophenol blue. The gels were run with a running buffer in a PROTEAN II XL multi-cell (Bio-Rad, Hercules, CA, USA) electrophoresis tank under 10 mA constant for 30 min, and then 30 mA until the tracking dye reached the bottom of the gels. Three representative gels per sample were used for further analysis.

### 3.5. Gel Staining and Image Analysis

The gels were fixed in 50% (*v*/*v*) ethanol and 10% (*v*/*v*) acetic acid for overnight, washed with Milli-Q water three times for 10 min each, and stained with Colloidal Coomassie Blue G-250 (CCB) solution for 12 h [1]. After rinsed with water, the gels were digitized with a calibrated scanner (UMAX Powerlook 2100 XL; UMAX, Taiwan), annotated, analyzed for spot number and spot volume using Image Master 2D Platinum Software (Version 6.0; Amersham Biosciences, Uppsala, Sweden). Three replicate gels were run for each of three different pooled pollen samples, and protein spots observed consistently in replicate gels were selected for further analysis.

### 3.6. In-Gel Digestion and Mass Spectrometry

After 2-DE, the protein spots were manually cut from the gels and rinsed twice with Milli-Q water, destained with 100 mM Na_2_S_2_O_3_ and 30 mM K_3_Fe(CN)_6_, dehydrated with 25 mM NH_4_HCO_3_ and 50% (*v*/*v*) acetonitrile (ACN), reduced with 10 mM DTT, alkylated with 55 mM iodoacetamide, and then completely dried under vacuum. Protein digestion was performed with trypsin (Mass grade, Promega, Madison, WI, USA) using a MassPREP protein digest station (Micromass, Manchester, UK) and incubated overnight at 37 °C. The resulting tryptic digests were then analyzed by a MALDI-TOF/TOF tandem mass spectrometer ABI 4800 proteomics analyzer (Applied Biosystems, Framingham, MN, USA). To acquire the mass spectra, 0.4 µL samples were mixed with equal volumes of matrix solution containing 0.5 M α-cyano-4-hydroxycinnamic acid (CHCA), 50% (*v*/*v*) ACN and 0.05% (*v*/*v*) trifluoroacetic acid (TFA) and spotted onto a MALDI plate. Spectra were acquired in the 800–4000 *m/z* range, analyzed by 4000 Series Explorer Software v3.5 (AB SCIEX, Foster, CA, USA) in batch-processing mode of MS/MS. The intensity peaks were detected on minimum S/N ratio ≥10 and cluster area S/N threshold ≥40 without smoothing and raw spectrum filtering. Peptide precursor ions corresponding to contaminants including keratin and the trypsin autolytic products were excluded in a mass tolerance of 0.5 Da.

### 3.7. Protein Identification and Allergen Prediction

The peptide mass data were uploaded on the Protein Pilot software v3.0 (Applied Biosystems, Framingham, MN, USA) and MASCOT search engine [43], and subjected to blast against *Populus* *trichocarpa* genome database v3.0, NCBI non-redundant protein database and Swiss-Prot database. The following parameters were used for database searching: trypsin as the proteolytic enzyme, allowing for one missed cleavage; carbamidomethylation of cysteine as a fixed modification; oxidation of methionine as a variable modification. All of the positive proteins were identified with a Mowse score greater than 60 and 95% confidence interval. The identified proteins were categorized by function according to data from Blast2GO [44]. The output GO terms were then slimmed in REVIGO and treemaps were produced [45]. Allergen was predicted using the SDAP [7] on the base of sequence similarity (>35%) between presently obtained proteins and reported allergen proteins and the presence of consecutive amino acids (at least eight) in the analyzed protein sequences compared to known allergen proteins [7], and the Allergen Online [6].

### 3.8. Microarray Data Analyses

The microarray data for various tissues were available at NCBI Gene Expression Omnibus (GEO) database [46]. The series accession numbers GSE21481 (for *P. trichocarpa*) were used for the tissue-specific expression analysis. Probe sets corresponding to selected genes were identified using the online Probe Match tool POParray. For genes with one or more probe sets, the median of expression values was considered. The expression values were normalized by the Gene Chip Robust Multiarray Analysis (GCRMA) algorithm followed by log transformation and average calculation.

### 3.9. RNA Extraction and Quantitative Reverse Transcription Polymerase Chain Reaction (qRT-PCR) ANalysis

For RNA isolation and qRT-PCR, the leaves, stems, roots and pollen of *P. tomentosa* were harvested, immediately frozen in liquid nitrogen and stored at –80 °C for further analysis. Total RNA was isolated using the RNeasy Plant Mini Kit (Qiagen, Hilden, Germany) with on-column treatment using RNase-free DNase I (Qiagen) according to the manufacturer’s instructions to ensure no genomic DNA contamination. First-strand cDNA was synthesized with approximately 1 µg of purified total RNA using the SuperScript III reverse transcription kit (Invitrogen, Carlsbad, CA, USA). qRT-PCR was performed in the LightCycler 480 Detection System (Roche, Penzberg, Germany) with two *PtoActin* genes as internal reference. The details of the primers used are listed in Table S2. The reaction mixture (20 µL) contained 10 µL 2× SYBR Green Real-time PCR Master Mix (TaKaRa, Dalian, China), 0.5 µM of each of the forward and reverse primers, and 2 µL of cDNA template. The amplification was completed with the following cycling parameters: 95 °C for 30 s; followed by 40 cycles at 95 °C for 5 s, 60 °C for 30 s; 60 °C for 60 s and 50 °C for 30 s. qRT-PCR was carried out in triplicates (technical repeats) to ensure the reproducibility of the results. The relative expression ratios were calculated from the threshold cycle according to the delta-delta CT method [47].

## 4. Conclusions

In summary, this study presents a comprehensive proteomic analysis and candidates for possible allergenic proteins in mature pollen of *P*. *tomentosa*. A total of 412 protein spots were isolated by 2-DE, and 159 different proteins were identified from 242 protein spots using MALDI-TOF/TOF MS/MS analysis. Furthermore, 27 proteins were identified as putative allergens, such as heat shock protein, enolase, pollen Ole e 1 allergen, thioredoxin and profilins, and their expression patterns across different tissues were analyzed based on an Affymetrix microarray data and qRT-PCR results. To our knowledge, this study is the first report on identification of possible allergenic proteins from *P. tomentosa* pollen. Further studies involving purification, recombinant protein expression, and epitope mapping of the identified putative allergens can be used as potential candidates for the development of hypoallergenic vaccines and innovative methods for immunotherapy and component-resolved diagnosis of *P. tomentosa* pollen allergy.

## Figures and Tables

**Table 1:** The basic information of predicted allergenic proteins in the pollen of *P. tomentosa*.

Spot No.	Gene ID	Protein Description	Amino Acids (aa)	Corresponding of Known Allergen				
				Allergen	Accession No.	Amino Acids (aa)	Bit Score	E Score
4/8/21	Potri.003G143600.1	Heat shock protein 70 (Hsp 70) family protein	666	Cor a 10	CAC14168	668	791.1	0.0
5/7	Potri.003G006300.1	Chloroplast heat shock protein 70-2	706	Cor a 10	CAC14168	668	322.5	5.0 × 10^−89^
9	Potri.010G205700.1	Heat shock protein 70 (Hsp 70) family protein	648	Cla h 5.0101	P40918	643	605.2	3.7 × 10^−174^
10/12/13	Potri.009G079700.1	Mitochondrial HSO70 2	682	Cor a 10	CAC14168	668	392.8	3.4 × 10^−110^
11	Potri.001G285500.1	Mitochondrial HSO70 2	683	Cor a 10	CAC14168	668	400.4	1.7 × 10^−112^
25/62/71/72/241	Potri.006G116800.1	Enolase	445	Hev b 9	Q9LEI9	445	583.5	5.6 × 10^−168^
31	Potri.001G087500.1	Heat shock protein 70 (Hsp 70) family protein	666	Cor a 10	CAC14168	668	816.5	0.0
43	Potri.009G098100.1	Granulin repeat cysteine protease family protein	508	Act d 1	AAA32629	380	237.7	7.0 × 10^−64^
48	Potri.002G189900.1	Aldehyde dehydrogenase 2B7	540	Cla h 10.0101	P40108	496	364.1	8.8 × 10^−102^
49/50/53/54/59/222	Potri.015G131100.1	Enolase	445	Hev b 9	Q9LEJ0	445	545.7	1.4 × 10^−156^
55	Potri.007G018000.1	Thioredoxin H-type 1	122	Tri a 25.0101	Q9LDX4	125	114.8	5.5 × 10^−28^
88	Potri.005G015100.1	Calreticulin 1a	419	Pen ch 31.0101	AAX45072	557	91.6	7.9 × 10^−20^
90	Potri.013G009500.1	Calreticulin 1a	360	Pen ch 31.0101	AAX45072	557	90.2	1.9 × 10^−19^
98/105	Potri.002G034400.1	NmrA-like negative transcriptional regulator family protein	308	Bet v 6.0102	AAG22740	308	353.3	5.3 × 10^−99^
106	Potri.017G102000.1	Malate dehydrogenase	412	Mala f 4	AAD25927	342	244.0	6.4 × 10^−66^
122/123	Potri.012G090900.1	Cysteine proteinases superfamily protein	363	Act d 1	AAA32629	380	238.2	3.6 × 10^−64^
145	Potri.008G056300.1	Triosephosphate isomerase	255	Tri a 31.0101	CAC14917	253	332.0	9.6 × 10^−93^
156	Potri.001G392400.1	Pollen Ole e 1 allergen and extensin family protein	161	Lyc e LAT52	CAA33854	161	118.4	7.8 × 10^−29^
159/160	Potri.011G111300.1	Pollen Ole e 1 allergen and extensin family protein	164	Lyc e LAT52	CAA33854	161	120.8	1.5 × 10^−29^
172/173	Potri.013G089200.1	HSP20-like chaperones superfamily protein	192	Cas s 9.0101	CAE46905	154	103.8	2.1 × 10^−24^
183	Potri.018G083500.1	Thioredoxin-dependent peroxidase 1	162	Cand b 2	AAA34357	167	105.4	6.6 × 10^−25^
194	Potri.016G024700.1	Calmodulin 6	149	Tyr p 24.0101	ACL36923	153	112.0	5.8 × 10^−27^
197	Potri.003G047700.1	Profilin 3	131	Hev b 8.0201	Q9M7N0	131	195.6	2.9 × 10^−52^
202	Potri.001G190800.1	Profilin 5	133	Hev b 8.0201	Q9M7N0	131	144.2	9.0 × 10^−37^
210	Potri.005G232700.1	Thioredoxin H-type 1	114	Tri a 25.0101	Q9LDX4	125	115.4	3.3 × 10^−28^
215	Potri.009G022300.1	Cystatin B	100	Act d 4.0101	AAR92223	116	69.1	2.4 × 10^−14^
217/218	Potri.006G093500.1	HSP20-like chaperones superfamily protein	140	Cas s 9.0101	CAE46905	154	151.4	7.6 × 10^−39^

## Supplementary Materials

The following are available online at www.mdpi.com/1422-0067/19/1/250/s1.

## Abbreviations

2-DE	Two-dimensional gel electrophoresis
ACN	Acetonitrile
CCB	Colloidal coomassie blue g-250
CHAPS	3-(3-cholamidopropyl) dimethylammonio-1-propane sulfonate
CHCA	α-cyano-4-hydroxycinnamic acid
DTT	Dithiothreitol
FDA	Fluorescein diacetate
GCRMA	Gene chip robust multiarray analysis
GEO	Gene expression omnibus
GO	Gene ontology
IEF	Isoelectric focusing
IPG	immobilized pH gradient
LC-MS/MS	Liquid chromatography coupled to tandem mass spectrometry
MALDI-TOF	Matrix-assisted laser desorption/ionization time-of-flight
MS	Mass spectrometry
*M*_W_	molecular weight
p*I*	isoelectric point
PMF	Peptide mass fingerprint
SDAP	Structural database of allergenic proteins
SDS-PAGE	Sodium dodecyl sulfate-polyacrylamide gel electrophoresis
TFA	Trifluoroacetic acid
