# Supplementary material for: Proteomic Analysis and Identification of Possible Allergenic Proteins in Mature Pollen of Populus tomentosa
Source: Int J Mol Sci. 2018 Jan 16;19(1):250. doi: 10.3390/ijms19010250 (PMC5796197; doi:10.3390/ijms19010250)
Supplement: Supplementary file 1 [file ijms-19-00250-s001.pdf]

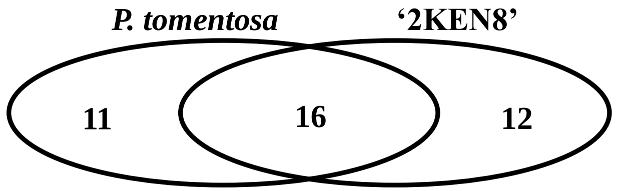

Spot No.	Gene ID	Protein description
5/7	Potri.003G006300.1	Chloroplast heat shock protein 70-2
10/12/13	Potri.009G079700.1	Mitochondrial HSO70 2
11	Potri.001G285500.1	Mitochondrial HSO70 2
25/62/71/72/241	Potri.006G116800.1	Enolase
31	Potri.001G087500.1	Heat shock protein 70 (Hsp 70) family protein
49/50/53/54/59/222	Potri.015G131100.1	Enolase
55	Potri.007G018000.1	Thioredoxin H-type 1
98/105	Potri.002G034400.1	NmrA-like negative transcriptional regulator family protein
145	Potri.008G056300.1	Triosephosphate isomerase
156	Potri.001G392400.1	Pollen Ole e 1 allergen and extensin family protein
159/160	Potri.011G111300.1	Pollen Ole e 1 allergen and extensin family protein
183	Potri.018G083500.1	Thioredoxin-dependent peroxidase 1
197	Potri.003G047700.1	Profilin 3
202	Potri.001G190800.1	Profilin 5
210	Potri.005G232700.1	Thioredoxin H-type1
217/218	Potri.006G093500.1	HSP20-like chaperones superfamily protein

Figure S1. The overlap of candidate allergenic proteins between *P. tomentosa* and "2KEN8". The Gene ID and protein description of 16 candidate allergenic proteins were showed in table.

Table S1. Proteins identified in mature pollen grains of *Populus tomentosa*.

Spot No.	Transcript name	PACid	Length (aa)	*Mw* (kDa) /p*I*	Score	Sequence coverage (%)	Protein description
Energy (56, 23.14%)							
15	Potri.017G136300.1	26984592	739	80.21/5.59	105	42	NADH-ubiquinone dehydrogenase, mitochondrial, putative
17	Potri.004G082800.1	26990681	736	79.96/5.90	59	38	NADH-ubiquinone dehydrogenase, mitochondrial, putative
22	Potri.007G026400.1	27017056	633	69.86/6.33	135	49	Succinate dehydrogenase 1-1
23	Potri.016G142900.1	27011564	633	61.18/5.40	278	40	Phosphoglycerate mutase, 2,3-bisphosphoglycerate-independent
24	Potri.017G136300.1	26984592	560	80.21/5.59	98	43	NADH-ubiquinone dehydrogenase, mitochondrial, putative
25	Potri.006G116800.1	27004351	739	47.64/5.56	175	60	Enolase
26	Potri.006G113300.1	27005580	445	61.01/5.37	265	35	Phosphoglycerate mutase, 2,3-bisphosphoglycerate-independent
30	Potri.008G009500.1	27036198	560	67.58/6.56	93	33	D-3-phosphoglycerate dehydrogenase
44	Potri.003G043900.1	26997314	637	58.38/7.60	78	24	Dihydrolipoamide acetyltransferase, long form protein
48	Potri.002G189900.1	27022343	539	58.84/6.11	269	43	Aldehyde dehydrogenase 2B7
49	Potri.015G131100.1	27019636	540	47.93/5.67	90	55	Enolase
50	Potri.015G131100.1	27019636	445	47.93/5.67	175	60	Enolase
51	Potri.008G009500.1	27036198	445	67.58/6.56	191	44	D-3-phosphoglycerate dehydrogenase
53	Potri.015G131100.1	27019636	637	47.95/5.67	184	71	Enolase
54	Potri.015G131100.1	27019636	445	47.95/5.67	362	69	Enolase
56	Potri.017G144700.1	26984288	445	51.61/5.78	342	61	UDP-glucose pyrophosphorylase 2
57	Potri.017G144700.1	26984288	469	51.61/5.78	456	62	UDP-glucose pyrophosphorylase 2
58	Potri.007G064000.1	27016782	469	39.70/6.76	102	43	Isocitrate dehydrogenase 1
59	Potri.015G131100.1	27019636	366	47.93/5.67	280	60	Enolase
61	Potri.005G154600.1	27028549	445	52.59/5.13	114	17	ATP synthase subunit alpha
62	Potri.006G116800.1	27004351	445	47.64/5.56	296	60	Enolase
64	Potri.008G084400.1	27036639	480	42.60/5.98	246	59	Phosphoglycerate kinase
71	Potri.006G116800.1	27004351	445	47.64/5.56	389	72	Enolase
72	Potri.006G116800.1	27004351	401	47.64/5.56	335	65	Enolase

Spot No.	Transcript name	PACid	Length (aa)	*M*w (kDa) /p*I*	Score	Sequence coverage (%)	Protein description
73	Potri.008G126600.1	27039927	445	59.97/5.91	66	39	ATP synthase alpha/beta family protein
74	Potri.016G028000.1	27011934	445	46.49/5.42	240	65	Regulatory particle triple-A ATPase 3
76	Potri.008G126600.1	27039927	559	59.97/5.91	618	67	ATP synthase alpha/beta family protein
77	Potri.008G126600.1	27039927	412	59.97/5.91	241	66	ATP synthase alpha/beta family protein
79	Potri.005G162100.1	27029430	559	20.98/4.44	187	49	Cytochrome C oxidase 6B
96	Potri.008G166800.1	27038013	559	42.90/7.07	413	55	Lactate/malate dehydrogenase family protein
97	Potri.002G141700.1	27024526	189	39.56/6.02	208	65	Lactate/malate dehydrogenase family protein
101	Potri.019G063600.1	27025643	392	34.95.5.81	458	65	PfkB-like carbohydrate kinase family protein
102	Potri.001G061400.1	27047655	364	34.95.5.81	146	41	Transketolase family protein
104	Potri.019G063600.1	27025643	327	34.95/5.81	298	65	PfkB-like carbohydrate kinase family protein
106	Potri.017G102000.1	26983417	367	43.32/8.15	417	64	Malate dehydrogenase
114	Potri.001G061400.1	27047655	327	39.39/5.87	404	53	Transketolase family protein
115	Potri.001G061400.1	27047655	412	39.39/5.87	243	41	Transketolase family protein
120	Potri.017G029000.1	26984355	367	35.26/4.93	344	60	PfkB-like carbohydrate kinase family protein
124	Potri.005G162100.1	27029430	367	20.98/4.44	130	46	Cytochrome C oxidase 6B
145	Potri.008G056300.1	27038997	328	27.36/6.00	633	61	Triosephosphate isomerase
155	Potri.003G086100.1	26999999	189	22.23/6.51	57	42	ATPase, F1 complex, delta/epsilon subunit
166	Potri.014G043300.1	27035250	255	22.79/5.56	219	59	UMP-CMP kinase
171	Potri.002G134600.1	27020771	207	22.08/5.45	268	67	UMP-CMP kinase
177	Potri.003G060100.1	26997045	205	26.82/6.00	149	41	Rubredoxin-like superfamily protein
180	Potri.010G217800.1	26980006	199	19.66/5.20	215	78	ATP synthase D chain, mitochondrial
183	Potri.003G086100.1	26999999	238	22.23/6.51	178	24	ATPase, F1complex, delta/epsilon subunit
193	Potri.001G173800.1	27046945	168	18.76/5.32	122	56	Cytochrome c oxidase-related family
207	Potri.013G108300.1	26995509	207	12.15/5.48	93	58	Vacuolar ATP synthases ubunit G2
212	Potri.002G062500.1	27024233	169	17.21/5.77	240	65	Related to ubiquitin
222	Potri.015G131100.1	27019636	108	47.93/5.67	109	56	Enolase

Spot No.	Transcript name	PACid	Length (aa)	*M*w (kDa) /p*I*	Score	Sequence coverage (%)	Protein description
225	Potri.001G456800.1	27047947	154	39.58/8.22	225	48	NADPH-dependent thioredoxin reductase A
226	Potri.019G063600.1	27025643	369	34.95/5.81	248	60	PfkB-like carbohydrate kinase family protein
228	Potri.019G063600.1	27025643	327	34.95/5.81	85	55	PfkB-like carbohydrate kinase family protein
232	Potri.008G009500.1	27036198	327	67.58/6.56	145	77	D-3-phosphoglycerate dehydrogenase
239	Potri.007G026400.1	27017056	637	69.86/6.33	203	59	Succinate dehydrogenase 1-1
241	Potri.006G116800.1	27004351	445	47.64/5.56	250	69	Enolase
Protein fate (43, 17.77%)							
3	Potri.005G120700.1	27028567	1462	165.86/5.53	49	22	Chaperone DnaJ-domain superfamily protein
4	Potri.003G143600.1	26996994	666	73.74/5.10	436	45	Heat shock protein 70 (Hsp 70) family protein
5	Potri.003G006300.1	26999807	706	75.34/5.24	492	37	Chloroplast heat shock protein 70-2
7	Potri.003G006300.1	26999807	706	75.34/5.24	437	48	Chloroplast heat shock protein 70-2
8	Potri.003G143600.1	26996994	666	73.47/5.10	436	45	Heat shock protein 70 (Hsp 70) family protein
9	Potri.010G205700.1	26979690	648	71.14/5.10	311	52	Heat shock protein 70 (Hsp 70) family protein
10	Potri.009G079700.1	26987596	682	73.26/5.56	82	37	Mitochondrial HSO70 2
11	Potri.001G285500.1	27041993	683	73.21/5.56	92	39	Mitochondrial HSO70 2
12	Potri.009G079700.1	26987596	682	73.26/5.56	174	38	Mitochondrial HSO70 2
13	Potri.009G079700.1	26987596	682	73.26/5.56	79	36	Mitochondrial HSO70 2
14	Potri.002G252900.1	27020402	575	61.12/5.84	132	56	Heat shock protein 60
21	Potri.003G143600.1	26996994	666	73.47/5.10	436	45	Heat shock protein 70 (Hsp 70) family protein
27	Potri.002G082100.1	27024556	505	56.18/4.76	261	50	PDI-like 1-2
28	Potri.002G082100.1	27024556	505	56.18/4.76	261	50	PDI-like 1-2
31	Potri.001G087500.1	27042356	666	73.51/5.05	530	47	Heat shock protein 70 (Hsp 70) family protein
32	Potri.002G082100.1	27024556	505	56.18/4.76	478	62	PDI-like 1-2
33	Potri.009G009300.1	26988421	586	61.99/5.24	66	39	Chaperonin-60alpha
35	Potri.001G002500.1	27044297	607	64.24/5.62	145	40	TCP-1/cpn60 chaperonin family protein
36	Potri.001G002500.1	27044297	607	64.24/5.62	88	42	TCP-1/cpn60 chaperonin family protein

Spot No.	Transcript name	PACid	Length (aa)	*M*w (kDa) /p*I*	Score	Sequence coverage (%)	Protein description
37	Potri.001G054400.1	27042250	574	60.99/5.66	93	54	Heat shock protein 60
38	Potri.001G054400.1	27042250	574	60.99/5.66	291	62	Heat shock protein 60
39	Potri.003G173900.1	26997267	574	61.18/5.75	275	59	Heat shock protein 60
41	Potri.003G173900.1	26997267	574	61.18/5.75	168	57	Heat shock protein 60
42	Potri.002G252900.1	27020402	575	61.12/5.84	139	59	Heat shock protein 60
55	Potri.007G018000.1	27015291	122	13.28/5.13	63	52	Thioredoxin H-type 1
107	Potri.014G122800.1	27032715	358	39.40/5.63	245	46	Thioredoxin family protein
110	Potri.002G198300.1	27022707	359	39.43/5.56	292	38	Thioredoxin family protein
111	Potri.002G198300.1	27022707	359	39.43/5.56	421	38	Thioredoxin family protein
112	Potri.014G122800.1	27032715	358	39.40/5.63	323	52	Thioredoxin family protein
128	Potri.018G063200.1	27008887	258	27.12/7.77	288	59	Chaperonin 20
136	Potri.006G138600.1	27006869	285	30.36/8.63	182	59	Chaperonin 20
146	Potri.014G122800.1	27032715	358	39.40/5.63	241	48	Thioredoxin family protein
151	Potri.018G145900.1	27009726	237	25.72/5.37	396	67	N-terminal nucleophile aminohydrolases (Ntn hydrolases) superfamily protein
153	Potri.014G069800.1	27033975	223	24.68/6.31	198	86	N-terminal nucleophile aminohydrolases (Ntn hydrolases) superfamily protein
163	Potri.003G109200.1	26999653	213	23.91/6.35	118	52	Mitochondrion-localized small heat shock protein 23.6
165	Potri.019G073700.1	27026328	308	34.49/5.83	259	17	Matrix metalloproteinase
172	Potri.013G089200.1	26994024	192	21.82/5.91	192	64	HSP20-like chaperones superfamily protein
173	Potri.013G089200.1	26994024	192	21.82/5.91	121	61	HSP20-like chaperones superfamily protein
179	Potri.010G217800.1	26980006	168	19.66/5.20	130	68	ATP synthase D chain, mitochondrial
205	Potri.010G217800.1	26980006	168	19.66/5.20	272	81	ATP synthase Dchain, mitochondrial
210	Potri.005G232700.1	27029465	114	12.58/5.57	126	47	Thioredoxin H-type1
217	Potri.006G093500.1	27006960	140	15.85/5.82	70	51	HSP20-like chaperones superfamily protein
218	Potri.006G093500.1	27006960	140	15.85/5.82	74	51	HSP20-like chaperones superfamily protein
Protein synthesis and processing (39, 16.12%)							

Spot No.	Transcript name	PACid	Length (aa)	*Mw* (kDa) /p*I*	Score	Sequence coverage (%)	Protein description
1	Potri.016G091600.1	27012288	802	89.33/5.07	234	44	ATPase, AAA-type, CDC48 protein
2	Potri.016G091600.1	27012288	802	89.33/5.07	274	50	ATPase, AAA-type, CDC48 protein
6	Potri.002G248300.1	27024631	588	65.70/5.14	138	33	Rotamase FKBP 1
16	Potri.009G020800.1	26987961	249	27.37/6.61	44	57	20S proteasome alpha subunit G1
20	Potri.002G248300.1	27024631	588	65.70/5.14	132	32	Rotamase FKBP 1
60	Potri.005G025100.1	27031369	423	47.39/4.94	214	63	Regulatory particle triple-A ATPase 5A
65	Potri.014G138100.1	27033304	450	49.06/6.44	242	58	GTP binding elongation factor Tu family protein
80	Potri.001G117900.1	27045733	147	16.56/4.76	43	72	Calcium-binding EF-hand family protein
84	Potri.013G016800.1	26995609	423	47.54/4.98	345	69	Regulatory particle triple-A ATPase 5A
103	Potri.015G003000.1	27018756	423	129.77/8.85	34	29	Nuclear RNA polymerase C2
122	Potri.012G090900.1	27050107	1157	40.42/5.80	152	25	Cysteine proteinases superfamily protein
123	Potri.012G090900.1	27050107	363	40.42/5.80	156	18	Cysteine proteinases superfamily protein
126	Potri.003G190800.1	26998519	363	22.46/4.33	349	60	Nascent polypeptide-associated complex (NAC), alpha subunit family protein
127	Potri.001G162900.1	27045481	205	26.03/4.76	393	72	20S proteasome alpha subunit E2
131	Potri.001G332700.1	27046848	237	24.32/5.24	86	40	Aldolase-type TIM barrel family protein
135	Potri.015G122400.1	27018307	226	31.70/8.95	117	47	Proteasome subunit PAB1
143	Potri.006G100500.1	27003778	288	33.06/6.25	139	47	Aldolase-type TIM barrel family protein
152	Potri.001G032900.1	27041096	309	22.35/5.76	289	72	Cystatin B
157	Potri.005G025100.1	27031369	246	47.39/4.94	555	65	Regulatory particle triple-A ATPase 5A
158	Potri.006G209600.1	27006760	200	46.87/7.74	34	39	Sorting nexin 1
161	Potri.002G062500.1	27024233	405	17.21/5.77	240	65	Related to ubiquitin
174	Potri.002G257500.1	27023000	140	15.01/10.48	60	47	60S ribosomal protein L23
176	Potri.008G092000.1	27039265	159	17.42/5.60	135	76	Eukaryotic elongation factor 5A-1
182	Potri.005G206300.1	27027956	145	15.45/5.70	344	65	40S ribosomal protein S12
184	Potri.002G056200.1	27022822	146	15.32/5.48	307	44	40S ribosomal protein S12

Spot No.	Transcript name	PACid	Length (aa)	*M*w (kDa) /*pI*	Score	Sequence coverage (%)	Protein description
185	Potri.003G126100.1	26996948	145	26.28/9.29	85	52	40S ribosomal protein S12
186	Potri.001G046600.1	27044522	238	15.08/5.63	92	39	40S ribosomal protein S12
187	Potri.001G046600.1	27044522	143	15.08/5.63	92	49	40S ribosomal protein S12
188	Potri.018G009100.1	27010285	143	21.76/11.36	33	33	40S ribosomal protein S12
189	Potri.018G009100.1	27010285	198	21.76/11.36	22	26	40S ribosomal protein S12
190	Potri.011G026600.1	27001472	198	25.65/6.86	220	59	Polyubiquitin family protein
192	Potri.001G046600.1	27044522	229	15.08/5.63	178	62	40S ribosomal protein S12
199	Potri.002G248200.1	27024141	143	16.06/7.68	70	70	Peptidyl-prolyl cis-trans isomerase
200	Potri.002G248200.1	27024141	153	16.06/7.68	70	69	Peptidyl-prolyl cis-trans isomerase
206	Potri.002G056200.1	27022822	153	15.32/5.48	310	57	Ribosomal protein L7Ae/L30e/S12e/Gadd45 family protein
215	Potri.009G022300.1	26988496	154	11.24/6.58	127	77	Cystatin B
223	Potri.006G008800.1	27005638	100	27.40/5.96	404	74	20S proteasome alpha subunit C1
220	Potri.006G140400.1	27005415	250	27.31/5.73	207	49	20S proteasome subunit PAA2
240	Potri.003G081000.1	26997593	232	25.32/4.53	233	48	Ubiquitin C-terminal hydrolase 3
Metabolism (30, 12.39%)							
45	Potri.003G072600.1	26997206	533	58.27/5.77	85	33	Alanine aminotransferase 2
52	Potri.006G123200.1	27003913	390	42.57/5.76	130	57	Methionine adenosyltransferase 3
63	Potri.011G136000.1	27002228	327	51.46/6.72	64	32	Tryptophan synthase beta-subunit 2
66	Potri.006G123200.1	27003913	472	42.57/5.76	430	60	Methionine adenosyltransferase 3
67	Potri.006G123200.1	27003913	390	42.57/5.76	430	60	Methionine adenosyltransferase 3
68	Potri.010G153500.1	26979918	390	43.18/5.50	208	52	S-adenosylmethionine synthetase 1
69	Potri.010G153500.1	26979918	395	43.18/5.50	336	63	S-adenosylmethionine synthetase 1
75	Potri.008G099300.1	27038006	395	43.17/5.50	453	62	S-adenosylmethionine synthetase family protein
92	Potri.010G153500.1	26979918	395	43.18/5.50	43	40	S-adenosylmethionine synthetase 1
93	Potri.017G099100.1	26985047	395	41.24/5.83	122	43	Reversibly glycosylated polypeptide 2
94	Potri.017G099100.1	26985047	365	41.24/5.83	407	56	Reversibly glycosylated polypeptide 2

Spot No.	Transcript name	PACid	Length (aa)	*M*w (kDa) /p*I*	Score	Sequence coverage (%)	Protein description
95	Potri.017G099100.1	26985047	365	41.24/5.83	489	71	Reversibly glycosylated polypeptide 2
109	Potri.012G045900.1	27050857	365	36.25/7.11	226	56	Galactose mutarotase-like superfamily protein
118	Potri.009G121300.1	26987713	327	43.66/5.76	89	19	Papain family cysteine protease
133	Potri.014G107100.1	27033421	396	24.87/5.69	186	39	Pyrophosphorylase 1
134	Potri.009G015400.1	26988032	216	27.40/6.71	236	66	Ascorbate peroxidase 1
139	Potri.014G107100.1	27033421	251	24.87/5.69	345	55	Pyrophosphorylase 1
140	Potri.009G015400.1	26988032	216	27.40/6.71	338	68	Ascorbate peroxidase 1
148	Potri.005G229000.1	27030087	251	29.36/6.32	192	46	Gamma carbonic anhydrase 1
149	Potri.006G082500.1	27005438	271	24.70/5.92	261	50	Pyrophosphorylase 4
154	Potri.012G045900.1	27050857	216	36.25/7.11	224	54	Galactose mutarotase-like superfamily protein
164	Potri.014G043300.1	27035250	205	22.79/5.56	251	80	P-loop containing nucleoside triphosphate hydrolases superfamily protein
167	Potri.002G134600.1	27020771	199	22.08/5.45	344	54	P-loop containing nucleoside triphosphate hydrolases superfamily protein
168	Potri.014G043300.1	27035250	205	22.79/5.56	210	75	P-loop containing nucleoside triphosphate hydrolases superfamily protein
170	Potri.002G134600.1	27020771	199	22.08/5.45	268	48	P-loop containing nucleoside triphosphate hydrolases superfamily protein
229	Potri.010G153500.1	26979918	395	43.18/5.50	329	57	S-adenosylmethionine synthetase 1
231	Potri.010G224300.1	26978976	355	39.11/5.23	257	44	Adenosine kinase 2
234	Potri.004G094600.1	26992574	577	61.70/5.83	54	42	Thiamine pyrophosphate dependent pyruvate decarboxylase family protein
237	Potri.014G168700.1	27032122	474	49.49/8.12	238	39	Acetoacetyl-CoA thiolase 2
238	Potri.017G099100.1	26985047	365	41.24/5.83	193	47	Reversibly glycosylated polypeptide 2
Defense/Stress response (17, 7.02%)							
34	Potri.010G175000.1	26981808	422	46.31/4.76	135	35	Metacaspase 4
40	Potri.010G175000.1	26981808	422	46.31/4.76	63	31	Metacaspase 4
43	Potri.009G098100.1	26986423	508	55.82/5.50	86	34	Granulin repeat cysteine protease family protein
46	Potri.012G143200.1	27050460	731	81.53/6.73	42	44	C2H2-like zinc finger protein
116	Potri.001G029900.1	27044936	136	15.34/9.30	44	60	LYR family of Fe/S cluster biogenesis protein

Spot No.	Transcript name	PACid	Length (aa)	*M*w (kDa) /p*I*	Score	Sequence coverage (%)	Protein description
125	Potri.015G057400.1	27018937	240	24.88/4.95	126	26	Glycine-rich RNA-binding protein 3
130	Potri.005G085500.1	27029144	249	28.96/8.44	340	60	Copper ion binding; cobalt ion binding; zinc ion binding
138	Potri.005G085500.1	27029144	249	28.96/8.44	258	53	Copper ion binding; cobalt ion binding; zinc ion binding
141	Potri.010G211600.1	26980673	228	25.50/7.86	299	75	Dehydroascorbate reductase 2
144	Potri.007G079500.1	27015019	240	27.84/8.50	438	61	Copper ion binding; cobalt ion binding; zinc ion binding
169	Potri.003G126100.1	26996948	238	26.28/9.29	177	60	Glutathione peroxidase 6
178	Potri.009G116400.1	26985817	170	17.00/5.54	86	51	Cold, circadian rhythm, and RNA binding 1
181	Potri.009G116400.1	26985817	170	17.00/5.54	328	50	Cold, circadian rhythm, and RNA binding 1
214	Potri.005G044400.1	27030154	162	15.36/5.60	123	60	Copper/zincsuperoxidedismutase 1
224	Potri.012G061600.1	27050381	152	26.43/4.84	216	34	Glycine-rich RNA-binding protein 3
227	Potri.018G083500.1	27009533	255	17.42/5.55	482	46	Thioredoxin-dependent peroxidase 1
236	Potri.005G071500.1	27031300	1743	195.85/7.64	38	18	Helicase domain-containing protein/IBR domain-containing protein/zinc finger protein-related
Development (13, 5.37%)							
70	Potri.004G189900.1	26990565	389	43.51/5.59	219	46	UDP-D-apiose/UDP-D-xylose synthase 2
85	Potri.019G090300.1	27026133	469	50.90/5.34	84	47	Late embryogenesis abundant domain-containing protein
87	Potri.007G024000.1	27015922	415	44.88/4.64	173	40	Late embryogenesis abundant (LEA) protein
89	Potri.017G092000.1	26985038	481	52.97/5.71	136	47	UDP-glucose 6-dehydrogenase
91	Potri.005G122400.1	27031280	408	44.32/4.81	175	43	Late embryogenesis abundant (LEA) protein
100	Potri.016G078300.1	27012190	362	38.94/6.23	436	53	Cinnamyl alcohol dehydrogenase 6
129	Potri.005G048000.1	27028208	263	27.71/5.26	71	45	Seed maturation protein
195	Potri.010G062800.1	26983086	91	9.69/5.51	109	43	Late embryogenesis abundant protein (LEA)family protein
196	Potri.001G172900.1	27047611	104	11.07/4.67	74	38	Late embryogenesis abundant protein (LEA) family protein
201	Potri.002G006000.1	27021334	91	9.95/5.12	74	38	Late embryogenesis abundant protein, group 6
213	Potri.002G252100.1	27023251	145	15.38/9.45	72	59	Late embryogenesis abundant domain-containing protein
230	Potri.007G024000.1	27015922	415	44.88/4.64	181	46	Late embryogenesis abundant (LEA) protein

Spot No.	Transcript name	PACid	Length (aa)	*M*w (kDa) /p*I*	Score	Sequence coverage (%)	Protein description
242	Potri.017G144700.1	26984288	469	51.61/5.78	219	46	UDP-glucose pyrophosphorylase 2
Cytoskeleton (5, 2.07%)							
18	Potri.004G073900.1	26992950	472	51.56/6.11	107	28	Pectin lyase-like superfamily protein
19	Potri.004G073900.1	26992950	472	51.56/6.11	107	28	Pectin lyase-like superfamily protein
197	Potri.003G047700.1	27000020	131	14.10/4.71	411	58	Profilin 3
202	Potri.001G190800.1	27047643	133	14.69/6.12	132	51	Profilin 5
235	Potri.017G099100.1	26985047	365	41.24/5.83	154	52	Reversibly glycosylated polypeptide 2
Cell fate (5, 2.07%)							
47	Potri.012G143200.1	27050460	731	81.53/6.73	42	44	C2H2-like zinc finger protein
82	Potri.019G102000.1	27024897	384	41.79/5.22	74	22	Clathrin light chain protein
83	Potri.019G102000.1	27024897	384	41.79/5.22	58	18	Clathrin light chain protein
88	Potri.005G015100.1	27027717	419	48.01/4.35	208	61	Calreticulin 1a
90	Potri.013G009500.1	26994433	360	41.46/4.65	152	50	Calreticulin 1a
Signal transduction (4, 1.65%)							
29	Potri.008G168100.1	27039956	463	52.44/9.78	48	36	Casein kinase I
99	Potri.002G095600.1	27020338	316	35.97/6.15	398	58	Annexin 1
108	Potri.001G174600.1	27041495	350	38.00/5.59	174	58	Transducin/WD40 repeat-like superfamily protein
147	Potri.015G068900.1	27018482	301	32.77/5.67	331	70	Transducin family protein/WD40 repeat family protein
Transport (2, 0.83%)							
208	Potri.012G039100.1	27050254	95	11.01/5.81	161	40	Tim 10/DDPfamilyzincfinger protein
209	Potri.012G039100.1	27050254	95	11.01/5.81	143	38	Tim 10/DDPfamilyzincfinger protein
Cell structure (1, 0.41%)							
113	Potri.006G192700.1	27008202	377	41.70/5.31	602	79	Actin-11
Unclassified protein (27, 11.16%)							
78	Potri.011G138400.1	27000938	451	49.83/7.19	273	36	RNA-binding protein 45A
81	Potri.007G124700.1	27015290	639	69.23/9.28	41	27	Similar to expressed protein

Spot No.	Transcript name	PACid	Length (aa)	*M*w (kDa) /*pI*	Score	Sequence coverage (%)	Protein description
86	Potri.007G018000.1	27015291	415	13.28/5.13	63	52	Similar to latex abundant protein 1
98	Potri.002G034400.1	27020856	308	33.98/5.51	280	45	NmrA-like negative transcriptional regulator family protein
105	Potri.002G034400.1	27020856	308	33.98/5.51	146	40	NmrA-like negative transcriptional regulator family protein
117	Potri.003G095400.1	26999460	277	29.54/5.42	97	38	RNA-binding (RRM/RBD/RNP motifs) family protein
119	Potri.T066800.1	27027243	430	47.19/5.25	204	37	RNA-binding (RRM/RBD/RNP motifs) family protein
121	Potri.009G073400.1	26988410	218	24.13/4.67	63	51	RAN binding protein 1
132	Potri.017G100000.1	26984684	245	27.57/8.23	157	52	Glutathione S-transferase
137	Potri.013G057700.1	26995766	342	37.83/6.67	118	39	Hypothetical protein POPTR_0013s05420g
142	Potri.008G102900.1	27037622	356	38.65/5.62	209	48	RmlC-like cupins superfamily protein
150	Potri.018G116300.1	27009432	212	22.89/12.21	50	57	Sserine-rich protein-related
156	Potri.001G392400.1	27041887	161	17.76/4.78	48	46	Pollen Ole e 1 allergen and extensin family protein
159	Potri.011G111300.1	27001602	164	17.88/4.85	213	53	Pollen Ole e 1 allergen and extensin family protein
160	Potri.011G111300.1	27001602	164	17.88/4.85	75	38	Pollen Ole e 1 allergen and extensin family protein
162	Potri.013G010500.1	26995700	899	99.79/8.66	41	36	Phosphatidylinositol N-acetylglucosaminyl transferase
175	Potri.T162000.1	27051474	134	14.87/5.84	143	47	Hypothetical protein POPTR_0323s00210g
191	Potri.016G023700.1	27012322	167	18.02/6.37	42	39	Histidine triad (HIT) family protein
194	Potri.016G024700.1	27014046	149	16.85/4.11	231	47	Calmodulin 6
198	Potri.T162000.1	27051474	134	14.87/5.84	143	49	Hypothetical protein POPTR_0323s00210g
203	Potri.014G152500.1	27033659	131	14.75/8.95	26	55	Hypothetical protein POPTR_0014s15070g
204	Potri.002G006000.1	27021334	91	9.95/5.12	74	38	Late embryogenesis abundant protein
211	Potri.010G236500.1	26978674	85	8.94/6.23	63	46	Copper chaperone
216	Potri.017G110500.1	26984818	345	38.51/6.33	31	39	NAD dependent epimerase/dehydratase
219	Potri.015G122500.1	27018687	155	16.81/4.79	52	32	Single hybrid motif superfamily protein
221	Potri.017G144700.1	26984288	469	51.61/5.78	179	36	Single hybrid motif superfamily protein
233	Potri.004G127400.1	26991750	415	46.47/4.66	396	27	similar to latex abundant protein 1

Table S2. Gene-specific primers used in qRT-PCR.

Gene ID	Protein description	Forward and reverse primers (5′–3′)	
Potri.010G205700.1	Heat shock protein 70 (Hsp 70) family protein	ACTACCTACTCATGCGTCGG	GGTTCATGGCGACCTGATTC
Potri.009G098100.1	Granulin repeat cysteine protease family protein	TGCTGTCCTCACGAGTATCC	AGCTGGTGAGACTGAGCAAT
Potri.002G189900.1	Aldehyde dehydrogenase 2B7	TTGAGAGTGGAGCGAACCTT	GCGACTGTTGTTGGACCTTT
Potri.007G018000.1	Thioredoxin H-type 1	AGAAGCTGGTGGTGATTG	CCACAACTTTGTCCACAATC
Potri.002G034400.1	NmrA-like negative transcriptional regulator family protein	ATTGGGAGCATGCAAATAGC	GCAGCAAAGAAGTTGGAAGG
Potri.008G056300.1	Triosephosphate isomerase	ATCTGGATATTCCCTGGGTC	TGCAGCAACAACCTCCACG
Potri.001G392400.1	Pollen Ole e 1 allergen and extensin family protein	CCAAAATCAGCGAGGGAATA	AGAATCAATGCTCCGGAATG
Potri.011G111300.1	Pollen Ole e 1 allergen and extensin family protein	TGTCGAAGGCAAGGTTTAC	TCGGCTCTGGGGCTCTCCAC
Potri.016G024700.1	Calmodulin 6	CGATGGTTGCATCACCACC	TGAGCTCCTCCTCGGAGTC
	PtoActin1	ACCCTCCAATCCAGACACTG	TTGCTGACCGTATGAGCAAG
	PtoActin2	ACTGTAATGGTCCTCCCTCC	CATCATCACAATCACTCTCC
